# Assessment of Technological and Sensory Properties, Digestibility, and Bioactive Compounds in *Polentas* from Different Maize Genotypes

**DOI:** 10.3390/foods13040590

**Published:** 2024-02-15

**Authors:** Nicolás Francisco Bongianino, María Eugenia Steffolani, Marianela Desiree Rodríguez, Mariela Cecilia Bustos, Carlos Alberto Biasutti, Alberto Edel León

**Affiliations:** 1 Córdoba Food Science and Technology Institute (ICYTAC), National Scientific and Technical, Research Council (CONICET), National University of Cordoba (UNC), Córdoba 5000, Argentina; nicolasbongianino@agro.unc.edu.ar (N.F.B.); eusteffolani@agro.unc.edu.ar (M.E.S.); mbustos@agro.unc.edu.ar (M.C.B.); 2 Plant Breeding, College of Agricultural Sciences, National University of Córdoba, CC 509, Córdoba 5000, Argentina; biasutti@agro.unc.edu.ar; 3 Biological Chemistry, College of Agricultural Sciences, National University of Córdoba, CC 509, Córdoba 5000, Argentina; 4 College of Agricultural Sciences, National University of Córdoba, CC 509, Córdoba 5000, Argentina; marianelarodriguez@agro.unc.edu.ar

**Keywords:** *polenta* digestion, antioxidant capacity, nutritional quality, healthy nutrition

## Abstract

The sensory profile of *polenta* and the connections between technological attributes and varieties of maize have not been extensively studied. Thus, it is necessary to understand the possible effect of its consumption on consumers’ health in terms of postprandial glucose levels and molecules associated with healthy activities. This work aims to study *polenta*’s technological and sensory properties from different maize genotypes and evaluate their digestibility and the potential contribution of bioactive compounds on health. A commercial hybrid, two open-pollinated varieties, and three inbred lines were used. Grain physical determinations and physical-chemical semolina traits were determined. *Polenta’s* technological quality was evaluated after simulated cooking. *In vitro* digestion was performed for *polentas*, and a sensory evaluation test was conducted. A significant correlation was found between semolina polyphenols and rapidly digestible starch (r = −0.6). Panellists characterised the genotype C6006 as having a good flavour, sandier mouthfeel, and low consistency. Also, the *polenta* from the hybrid exhibited sensory attributes more closely resembling commercial *polenta* in terms of maize odour, flavour, and consistency. The higher content of polyphenols presents in semolina affected the digestion of *polenta*, showing a lower proportion of rapidly digestible starch and a lower amount of bioaccessible protein fraction.

## 1. Introduction

Maize (*Zea mays* L.) is a cereal grain cultivated all over the world, and it is also widely used for human nutrition because it is adequate for the production of food, such as *polenta* [1]. *Polenta* is a dish made predominantly with maize grits, water, and salt. Cooking requires constantly stirring the mixture over low heat. This food is popular in northern Italy, other European countries, and South America [2]. 

Italian maize *polenta* was historically obtained from local varieties with flint or semi-flint kernels and hard, vitreous endosperms [3]. In Italy, as in other countries, after the Second World War, these traditional native varieties were replaced by high-yielding, toothy hybrids from the United States [4]. Argentina is one of the countries where the massive adoption of high-yielding dent hybrids occurred. These new cultivars are characteristically less suitable for dry milling than traditional Argentinean flint genotypes due to a higher number of floating grains, a lower fraction of vitreous endosperm, and lower test weight [5].

Traditionally, this food has been made from yellow, white, or red maize grains, which are dried and ground to obtain finer or coarser flours, depending on the region and desired texture [6]. The nature of starch fractions has an impact on *polenta* viscosity, as there is a negative correlation between grain hardness and flour viscosity properties [6,7]. However, studies on the chemical composition of grits of varying particle sizes have revealed that moisture content ranges between 10.01% and 12.43%, protein content from 7.05% to 8.31%, ash from 0.40% to 0.51%, fat from 3.40% to 5.51%, and total carbohydrate content between 75.39% and 76.75% [8]. The interactions between these components influence physicochemical and nutritional properties such as texture, taste, palatability, and the rate and extent of digestion/absorption. In addition, these interactions influence physiological characteristics, like postprandial blood glucose [9].

In cereal-based food, variations in starch status and differences in formulations and matrices trigger fluctuations in the degree and rate of starch hydrolysis through digestion [10]. Thus, starch digestibility and glycaemic index in cooked *polentas* are influenced by grain hardness, which is closely related to the composition of specific zeins and their ability to form a disulphide bond [11]. 

Considering genetic diversity in maize is important in terms of phytochemical compounds or secondary metabolites, such as carotenoids and phenolic compounds. Consumption of these compounds is associated with a lower risk of chronic diseases like cancer or diabetes [12]. Conversely, botanical polyphenols can interact with digestive enzymes and starch, generating a reduction in its hydrolysis. In this sense, consumers’ health can benefit from the reduction of the glycemic index [13]. Similarly, some authors [14] mentioned that maize polyphenols have antioxidant and anti-inflammatory properties and that these compounds are linked to potential benefits in Alzheimer’s disease. Correspondingly, some authors showed that when maize proteins were hydrolysed, there was a surge of bioactive peptides with inhibitory activity of enzymes involved in carbohydrate metabolism (α-amylase, α-glucosidase) [15].

According to some authors, the traditional raw material used for making *polenta* (flint grains) results in a low viscosity and consistency product, but it is also possible to select appropriate conventional hybrids for the *polenta* industry by performing quick measurements of the grain, despite the texture and colour differences [16]. However, the sensory profile of *polenta* and the connections between technological attributes and varieties of maize have not been extensively studied [2]. Thus, it is necessary to understand the possible effect of its consumption on consumers’ health in terms of postprandial glucose levels and molecules associated with healthy activities. This work aims to evaluate the physicochemical characteristics of maize grits from different genotypes and, after cooking the *polenta*, to study their technological and sensory properties, their behaviour for *in vitro* digestion, and the potential contribution of bioactive polyphenols to health.

## 2. Materials and Methods

### 2.1. Genetic Material

A commercial yellow soft-grain hybrid (H) (“AX882”), two open-pollinated varieties, (OPV) “C6006” and “C8008” (orange and white grains colour, respectively), and three inbred lines (L) (Yellow “B4”, White “C4B”, and White “BCOT”) were used. The OPV and L genotypes were developed at the Plant Breeding Department of the Faculty of Agricultural Sciences, National University of Cordoba (FCA—UNC).

### 2.2. Grain Physical Properties

The weight of 100 grains (W100) was determined using an analytical balance following the methodology of [17]. The test weight (TW) was estimated using a Schopper balance and a 250 mL container [18]. Grain hardness was determined indirectly by flotation Index (FI) in a sodium nitrate solution (density (q) 1.250 ± 0.001) following the method described by [19].

### 2.3. Maize Milling

The whole grain was cut using a butt mill (Decalab, Ciudad Autónoma de Buenos Aíres, Argentina). Then, the broken grains were milled in an Agromatic AG AQC 109 (Agromatic AG, Laupen, Switzerland). After that, the maize flour was passed through a sieve ZONYTEST EJR 2000 (Rey & Ronzoni, Ciudad Autónoma de Buenos Aíres, Argentina) for 20 min. The coarse flour fraction greater than 297 μm was considered semolina for *polenta*. The samples were stored in airtight plastic bags at 4 °C until determinations were made [16].

### 2.4. Physico-Chemical Semolina Traits

The proximal composition was determined through protein, lipids, ash, and starch content according to methods 46-10.01, 30-25.01, 08-01.01, and 76-13.01, respectively (AACC Approved Methods of Analysis, 2010). All determinations were expressed in g per 100 g of sample on a dry basis and performed in duplicate. The particle size distribution was obtained by the laser diffraction method using a particle size analyser LA 960 (HORIBA, Fukuchiyama, Japan). Particle size distribution was recorded for 90% or more of the sample in μm (D90). The span was calculated, which provided information on the amplitude and heterogeneity of particle size distribution: span = (D90 − D10)/D50.

### 2.5. Polenta Technological Quality

*Polenta* cooking simulation was made by a Rapid Visco Analyzer (RVA-4500, Perten Instruments, Springfield, IL, USA), and pasting properties were recorded [16]. Distilled water (20 g) was added to the ground maize (5 g) in an RVA aluminium canister (adjusted to 14% humidity). The cooked sample was stored at 7 °C for 24 h. To determine the force required to penetrate the *polenta*, a texture machine (Instron, High Wycombe, UK) was used. The machine had a 500 N cell, a 25 mm diameter probe, and a 50% deformation. The machine generated a force of 10 mm/s at an ambient temperature of 24 °C. The consistency values were expressed in degrees of force (gf).

The colour of cooked *polenta* was evaluated using a spectrophotometer CM-508d (Konica Minolta, Ramsey, NJ, USA) according to method 14-22.01 (AACC Approved Methods of Analysis, 2010). The Commission International de l’Elcairage (CIE) system coordinates were used [20].

### 2.6. Polenta In Vitro Digestion

*In vitro* digestion of *polenta* samples was performed in duplicate, according to the method of other authors [10], by taking aliquots of 1 mL at times 0, 2 (oral phase), 10, 30, 60, and 120 min of the gastric step, and 10, 30, 90, and 180 min of the intestinal phase. For dialyzability determinations, the assay was performed by introducing a dialysis membrane at the start of the intestinal phase with 25 mL of NaHCO_3_ equivalent to the titratable acidity (measured previously). Finally, the quantity of dialyzable starch fraction was determined to assess the potentially bioavailable fraction. In addition, an index was developed from the area under the starch hydrolysis curve (AUC index) using Equation (1) because this approach is particularly useful, as it allows a simple comparison of the *in vitro* glycemic response of each food with a reference food *in vivo*. To this end, a control food (white bread) was used to compare samples using a reference value of 100%. Thus, AUCs is the area under the curve of each sample and AUCwb is the area under the curve of the white bread used as a control.

AUC index = (“AUCs”/”AUCwb”)”×100”(1)

The process of starch hydrolysis was monitored during the experiment by analysing the content of reducing sugar in each aliquot using the 3-5 dinitrosalicylic acid (DNS) method. Two non-linear models, namely Equations (2) and (3), were used to describe the oral–gastric and intestinal digestion stages of starch hydrolysis, respectively. The estimation of parameters was performed with the SIGMA PLOT software, version 12. The rate of starch digestion was expressed as a percentage of the total starch present in each sample that underwent hydrolysis at different times.

Oral–gastric *in vitro* digestion: Cg = Cg ∞ × (1 − e − kg t) (2)

Intestinal *in vitro* digestion: Ci = C0 + Ci ∞ × (1 − e − ki t) (3)

The degree of starch hydrolysis during digestion, represented by the percentage of starch hydrolysed at a given time (C), is a function of the percentage of starch hydrolysed at an infinite time (C∞), the kinetic constant (K), and the percentage of starch hydrolysed at the beginning of the intestinal phase (C0). Parameters for oral–gastric digestion are denoted as “g”, while those for the intestinal phase are denoted as “i”. Starch is classified based on its rate of hydrolysis. Rapidly digestible starch (RDS) is digested within 20 min, slowly digestible starch (SDS) is digested between 20 and 180 min, and resistant starch (RS) remains undigested after 180 min.

### 2.7. Polyphenols Extraction and Quantification

Polyphenols extraction from semolina and lyophilized *polenta* was performed according to authors [21] with 1.5 mL (1:10) of ethanol (96%) /HCl (1 N) (85:15, *v*/*v*). The samples were centrifuged at 8000× *g* for 10 min and the supernatant was recovered. The extraction was repeated six times, which enabled us to save the supernatants. Polyphenol content was determined on semolina and *polenta* extract and in *polenta* fractions at the end of digestion (internal and external fraction of the dialysis membrane) by the Folin–Ciocalteu procedure and it was expressed as mg gallic acid equivalent per 100 g sample (mg GAE/100 g).

### 2.8. Antioxidant Capacity

Ferric Reducing Antioxidant Power (FRAP) activity was measured according to the published method [22] with some modifications. FRAP reagent was made using acetate buffer (300 mM, pH 3.6), TPTZ (2,4,6-tripyridyl-s-triazine) 20 mM in 40 mM HCl, and FeCl_3_·6H_2_O (20 mM). These components were mixed at 10:1:1. The isolated polyphenols (semolina and *polenta*) and the *polenta* fractions from the end of digestion (bioaccessible fractions) (100 µL) were mixed with 3 mL of working FRAP reagent. The samples were incubated at 37 °C for 30 min and absorbance was measured at 593 nm. Trolox solution was used as a standard for the calibration. The equivalent parameter concentration is mg Trolox per 100 g dry sample.

### 2.9. Protein Digestion

Protein hydrolysis was measured by the OPA method [23] for the initial process (T0) and in the fractions resistant to digestion. These fractions were formed by the content that remains outside the dialysis membrane (non-dialysable) and the fraction that passes through it (dialysable), which was considered a potentially bioavailable fraction. The serine standard was prepared as follows: 50 mg serine (Art. 7769 Merck, Darmstadt, Germany) was diluted in 500 mL deionized water (0.9516 meqv/L). All spectrophotometer readings were performed at 340 nm using deionized water as the control.

### 2.10. Polenta Sensory Quality

The sensory analysis was carried out using as a basis some descriptors published by other authors [6] and then adapted according to previous pilot tests. The selected samples included a typical *polenta* from flint-grain semolina (C6006), a *polenta* from the commercial hybrid (AX882), and a *polenta* with a distinct colour (white) and the highest antioxidant capacity in the bioaccessible digestion fraction (C8008). *Polenta* from commercial semolina was used as a middle point (4-point scale) for sensory descriptors. A group of semi-trained evaluators, consisting of thirteen men and fourteen women between the ages of twenty and sixty-six, participated in the study. The test was performed according to ISO 13299:2016, 8589:2010, 13300-1:2006, 3972:2011, and 5496:2006 (International Organization of Standardization Methods). *Polenta* descriptors were evaluated using a seven-point rating scale, and commercial *polenta* used as a control was placed on the medium scale (4). Descriptors included the following: Mo, maize odour (1—no maize odour and 7—very strong maize odour); F, flavour (1—not at all pleasant and 7—very pleasant); ML, (1—very sandy and 7—very creamy); C, consistency (1—very liquid and 7—very firm); A, adhesiveness (1—very little adhesiveness and 7—very adhesive); Cr, colour (1—not at all pleasant and 7—very pleasant); Su, surface homogeneity (1—not at all homogeneous and 7—very homogeneous). Finally, the preference order was evaluated.

### 2.11. Statistical Analysis

The statistical analyses were conducted using the InfoStat software (InfoStat, Cordoba, Argentina, Version 2020). To assess the power and direction of the linear relationships between the variables, the Pearson’s correlation test was used (*p* < 0.05 and 0.01). To compare the trait’s average values and ascertain if there are statistical differences between genotypes, an analysis of variance was performed with the LSD Fisher comparison test (*p* < 0.05).

## 3. Results and Discussion

### 3.1. Grain and Semolina for Polenta Physical-Chemical Traits

The inbred line C4B had the lowest 100-grain weight (23.72 g) (Table 1). Likewise, C4B (5.75%), BCOT (14.50%), and C6006 (21.50%) had the lowest FI values, while the commercial hybrid had the highest number of floating grains (90.5%). As for TW, the hybrid had the lowest value compared to the other genotypes, except for C8008, with which it was statistically equal. Moreover, AX882 had a smaller particle size than the inbred lines B4 and BCOT. Furthermore, it had a lower expression of span than C4B concerning semolina characteristics. The findings were consistent with those disclosed by authors [24], who examined the dent, semi-dent, and flint Brazilian genotypes. Thus, they showed similar values for floaters and different endosperm fractions, where hard grain genotypes had fewer floaters and a harder endosperm fraction than soft (dent) grain genotypes. Also, other authors [25] showed that soft endosperm genotypes tended to exhibit the lowest values of kernel density, grit yield, and milling ratio.

Concerning semolina chemical composition and cooking traits, the inbred lines C4B, B4, and BCOT showed the highest protein content (11.67, 11.48, and 10.82%, respectively), and H exhibited the lowest expression. Also, the genotype BCOT had the lowest value for the majority grain component (total starch) expression against C4B, B4, AX882, and C6006. Peak viscosity and *polenta* consistency were represented with the highest expression through the commercial hybrid, showing values of 5832.67 cP and 4301.99 gf, respectively (Table 1). Regarding *polenta*’s colour, statistical differences (*p* ≤ 0.05) were found. In this sense, the OPV C8008 reached lower a* and b* values than the other genotypes (white *polenta*). On the other hand, *polenta* from C6006 showed the highest value for a* and *polenta* from B4 obtained the highest b* value (Table 1). Some authors suggest that grain hardness is connected to *polenta* cooking traits. This means that genotypes with a soft endosperm have less compact cell bodies, which allows for a high degree of starch granule hydration and swelling during the cooking process. As a result, these types of grains produce *polenta* with a high viscosity and a consistent texture [16].

### 3.2. Static In Vitro Digestion

Starch hydrolysis showed statistical differences between genotypes (Table 2). In this sense, all samples (except BCOT) showed an RDS values lower than the white bread control. Also, the inbred line C4B had the lowest RDS value (approx. 56%) and an SDS value higher than the other samples with 30%. Conversely, the OPV C6006 showed the lowest SDS value (2%) and the highest proportion of resistant starch together with B4 with values of 28% and 30%. Some authors showed similar values for RDS (56.0 to 67.7%) and RS (28.9 to 38.4%), and a little difference for SDS (2.6 to 7.3%). Also, they affirmed that maize genotypes with hard endosperm exhibit high concentrations of two specific zeins (C1 and E zein) [26]. Even though this work did not extensively study corn proteins, it is crucial to note that these compounds create numerous disulphide bonds that mechanically limit the access of amylases to starch, reducing its hydrolysis. The ensuing outcome is a strong, negative correlation between rapidly digestible starch and the C1 zein group [11]. In addition, variations in the food matrix and structure have a significant impact on the hydrolysis process that takes place during oral digestion, ultimately influencing the entire digestive process [10]. 

The B4 inbred line had the lowest value of the AUC index (86.36%), and it was the only one below the control white bread (100%). Thus, this aspect needs to be considered carefully, as both the rate and extent of starch digestion impact various physiological and health functions, including reduced caloric value, hypocholesterolaemia, and protective effects against colorectal cancer [27].

**Figure 1:**
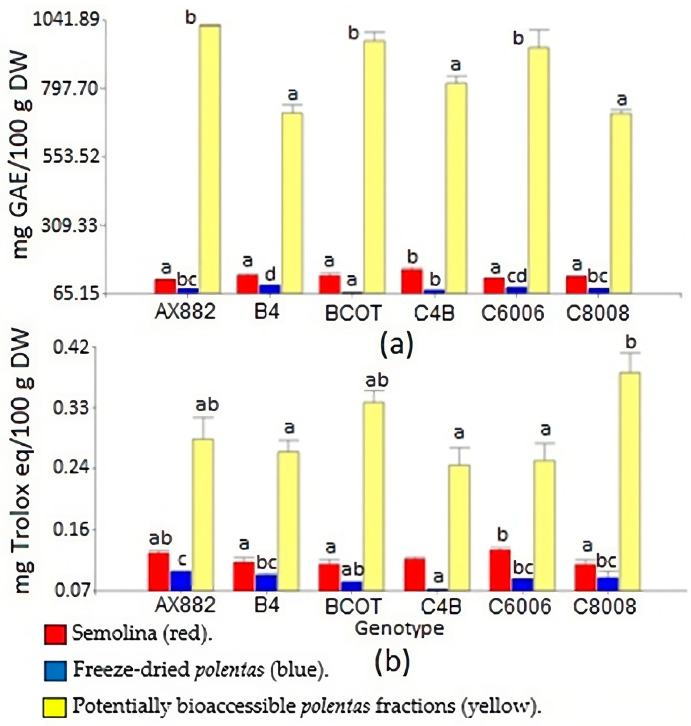
Polyphenols content (**a**) and antioxidant capacity (**b**). Different letters indicate statistical differences (LSD Fisher, *p* < 0.05).

Polyphenol content of genotypes (Figure 1a) showed differences (*p* < 0.05) among the three types of analysed samples: semolina, freeze-dried *polenta*, and potentially bioaccessible fraction. In general, we noted that cooking resulted in a decline of polyphenol levels across all the samples. This observation is consistent with the research conducted by authors [28], who reported a substantial drop in the total phenolic content after cooking. This decline was influenced by cooking conditions involving exposure to hot water and oxygen. In this sense, the inbred line C4B showed the highest value for semolina (150.54 mg GAE/100 g DW). The BCOT line reached the lowest polyphenol content in *polenta* (68.11 mg GAE/100 g DW), while the *polenta* from the B4 line achieved the highest value (94.31 mg GAE/100 g DW). Thus, our results showed that the *polenta* obtained from the B4 inbred line was one of the two samples with the highest RS content (mentioned above) and the highest presence of polyphenols after cooking. Regarding bioaccessible polyphenols, the genotypes AX882, BCOT, and C6006 showed high values over the rest of the samples. The antioxidant capacity of those samples also showed variations (Figure 1b). Although OPV C8008 is not the genotype with the highest antioxidant capacity in semolina and *polenta*, it showed the highest value in the bioaccessible fraction with 0.38 mg Trolox eq/100 g DW. The behaviour observed is consistent with research [29], which observed increased total phenolic content and antioxidant capacity of the samples when subjected to digestion. This is because the enzymatic action leaves a greater number of phenolic compounds available from the division of major phenolic structures and structural components of foods. 

Three correlations between polyphenols in semolina and grain-specific physical attributes derive from the results. Data showed that semolina polyphenols are related to W100 (*p* = 0.04, r = −0.59), TW (*p* = 0.02, r = 0.66), and FI (*p* = 0.04, r = −0.6). Some authors [30] suggested that the high concentration of phenolic acids may contribute to the hardness of grains due to their ability to cross-link with cell walls in the pericarp and aleurone layers. Thus, they showed that maize bran of harder grains had higher phenolic acid content than soft types According to the authors [31], there exists a significant correlation among antioxidant capacity and overall phenolic content, thermal properties, amylose content, and crystallinity in different types of maize germplasm. In this way, these findings help identify accessions with desired traits for both food and non-food use. During research on various types of nixtamalized maize for pozole, it was observed that there exists an inverse relationship between the softness of the grain and the levels of phenolic compounds and antioxidant capacity [32]. A significant correlation was found between semolina polyphenols and RDS (*p* = 0.04, r = −0.6). Research [33] showed that the digestion of starch can be impacted by polyphenols through several mechanisms. One such mechanism involves the accessibility of α-amylase and α-glucosidase enzymes, which can be influenced by phenolic compounds. Furthermore, supramolecular complexes can form between phenolic compounds and starch, contributing to this inhibitory effect.

Our results showed that *polenta* antioxidant capacity exhibited a significant Pearson correlation with the grain physical traits, namely W100 (*p* = 0.0012, r = 0.82), TW (*p* = 0.0043 and r = −0.76), and FI (*p* = 0.0028 and r = 0.78). As noted in previous research [34], the impact of antioxidants on the body depends not only on their concentration but also on their bioaccessibility and bioavailability following digestion. These factors are influenced by the degree to which these compounds are released from the food matrix. 

Concerning the relationship between the colour *polenta* and its characteristics, significant and negative correlations were found for L* with polyphenol content in the *polenta* and with the antioxidant capacity of this food, with values of *p* = 0.03 and r = −0.63 in both cases. These results coincide with research [35], which proved the same relationships among the lightness of the food, phenolic compounds, and its antioxidant capacity in maize tortillas. Conversely, semolina’s antioxidant capacity was related to significant, and positive effects of the colour a* element (red tone) and showed an r coefficient of 0.6 (*p* = 0.04). In this sense, pigmented maize contains carotenoids and phenolic compounds, and interest in its antioxidant and bioactive properties has increased due to their health benefits [36]. For their part, some authors [37] showed positive correlations between flour colour (a*) and provitamin A carotenoids with an r coefficient of 0.36. Other interesting relationships were found between the colour parameters and the percentage of resistant starch, where the L* trait was negatively associated with this starch fraction (*p* = 0.03 and r = −0.64). Furthermore, both the yellow (b*) and red tones of the samples (a*) presented a positive relationship with RS with r coefficients of 0.84 (*p* = 6.5 × 10 − 4) and 0.79 (*p* = 2.3 × 10 − 3), respectively. The use of pigmented maize flour has been identified as an effective strategy to develop healthier gluten-free food products. This is due to their remarkable antioxidant properties and higher levels of SDS and RS [28]. In general, flour from the flint grain type has higher a* and b* values and a lower L* compared to that from the dent [38]. By the same token, some authors have found that maize grains with higher hardness exhibit lower starch digestibility and a higher proportion of RS [11]. 

**Figure 2:**
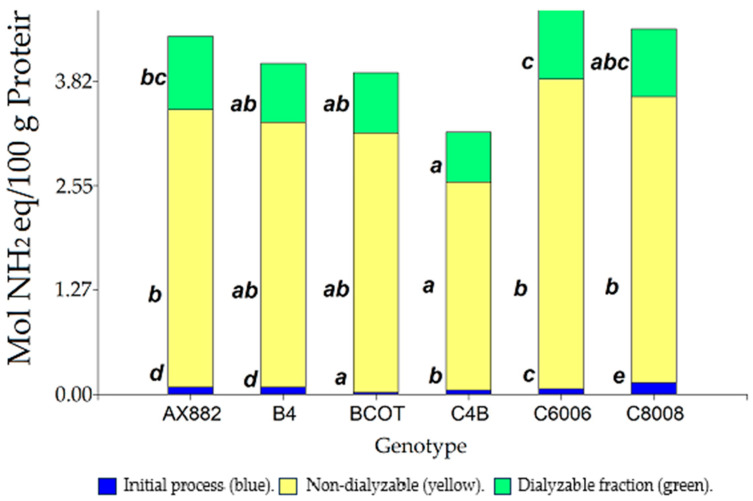
Protein hydrolysis of *polentas.* Different letters indicate statistical differences (LSD Fisher, *p* < 0.05).

Regarding protein digestion, genotypes showed variability among themselves both in the initial process (free amino acids) at T0 and in the fractions resistant to digestion (Figure 2). In this sense, the genotype C4B presented the lowest total protein hydrolysis (3.09 Mol NH2 eq/100 g protein) against C8008, AX882, and C6006. On the other hand, C6006 showed more potentially bioavailable protein than other samples (C4B, B4, and BCOT), with an average of 1 Mol NH2 eq/100 g protein reached inside the dialysis membrane. Some authors [39] showed that maize with provitamin A (orange grains colour) had a higher protein digestibility than other maize varieties. On the other hand, [40] research showed that maize tortillas with a lower proportion of β structures (β-sheet and β-turn) and lower content of α-helix had a higher protein digestibility. Additionally, these studies mentioned a strong negative linear correlation between the content of β conformations and *in vitro* protein digestibility. The digestion-resistant protein fraction (nondialysable and dialysable) presented a significant positive relationship with RDS (*p* = 0.05, r = 0.57) and a negative association with semolina polyphenols (*p* = 0.01 and r = −0.74). The intricate nature of protein digestion in cereals can be attributed to the formation of protein particles that remain insoluble in water due to the hydrophobic properties of zein. This insolubility can hinder the access of digestive enzymes, leading to reduced protein digestibility [41]. Additionally, polyphenols present in cereals can bind to digestive enzymes, further impeding protein breakdown and nutrient absorption [42].

### 3.3. Sensory Analysis

**Figure 3:**
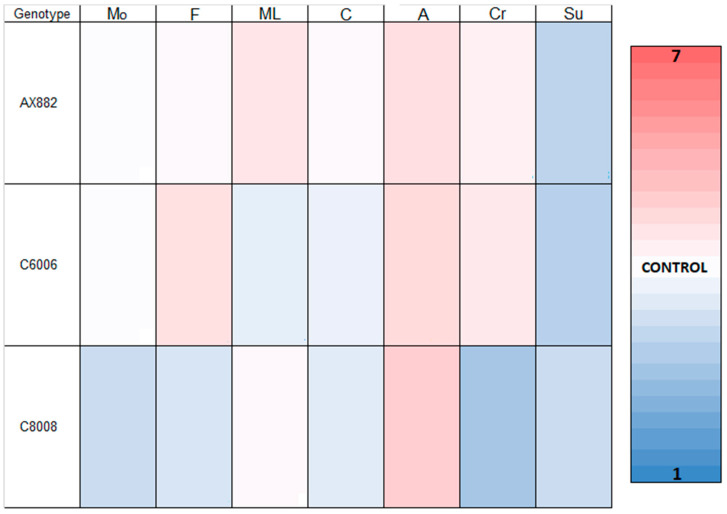
Sensory quality results. The seven-point rating scale used with commercial *polenta* on the medium scale (4). Descriptors included: Mo, maize odour (1—no maize odour and 7—very strong maize odour); F, flavour (1—not at all pleasant and 7—very pleasant); ML, (1—very sandy and 7—very creamy); C, consistency (1—very liquid and 7—very firm); A, adhesiveness (1—very little adhesiveness and 7—very adhesive); Cr, colour (1—not at all pleasant and 7—very pleasant); Su, surface homogeneity (1—not at all homogeneous and 7—very homogeneous).

The sensory analysis revealed variability between genotypes (Figure 3), where the OPV C6006 was characterised by panellists with a good flavour, sandier mouthfeel, and low consistency. The sandy sensation in the mouth is said to be directly related to the size, superficial homogeneity, and hardness of the food particles [43]. Foods with smaller and softer particles exert less deformation on the oral mucosa, making them less perceivable as distinct particles [44]. Conversely, *polenta* derived from the hybrid AX882 exhibited closer sensory attributes to the control commercial *polenta*, with maize odour, flavour, and consistency values approaching 4 and white colour. The possible explanation for this phenomenon is that a vast majority of Argentina’s maize production consists of semi-dentate grains with soft endosperms comprising high-yield commercial hybrids [45]. Contrary to authors [3], who showed that there were no differences in taste perception between coloured and uncoloured *polenta* as reported by consumers, the *polenta* from white OPV C8008 showed poor sensory traits, such as flavour and colour, compared to other *polenta* samples. These results were mirrored in a preference ranking, with *polenta* C6006 receiving the highest scores and *polenta* C8008 receiving the lowest scores in consumer preference tests. According to the descriptors outlined by other authors [6], our research revealed that the parameters in question play a critical role in distinguishing sensory qualities between samples, thus influencing overall preference.

## 4. Conclusions

This work’s findings suggest that grains with higher densities, harder textures, and lower weight per 100 grains are linked to higher semolina polyphenol content, which impacts *polenta* digestion. This suggests that these materials will contain less rapidly digested starch. Concerning the sensory preference of the samples evaluated, results may indicate that this is linked to a lower consistency and a more pleasant flavour and colour. It should be noted that the sample with the highest preference (OPV C6006) differed in several attributes from the other samples and the commercial control. In this regard, this genotype provided a *polenta* with a reddish hue and high levels of digestive traits, including resistant starch content, potentially bioaccessible polyphenols, and potentially bioavailable protein. Consequently, it is evident that consumers have a preference for traditional flint materials in the preparation of *polenta*.

## Figures and Tables

**Table 1:** Grain and semolina for *polenta* physical traits, chemical composition, and technological average values.

Genotype	AX882	B4	BCOT	C4B	C6006	C8008
W100 (g)	35.27 ± 4.79 ^bc^	36.89 ± 0.17 ^bc^	31.53 ± 0.30 ^b^	23.72 ± 0.39 ^a^	32.95 ± 1.41 ^bc^	38.24 ± 3.56 ^c^
TW (Kg/HL)	85.00 ± 2.26 ^a^	88.90 ± 1.84 ^bc^	93.40 ± 0.28 ^de^	95.50 ± 0.14 ^e^	92.00 ± 0.00 ^cd^	86.10 ± 0.71 ^ab^
FI (%)	90.50 ± 3.42 ^e^	47.50 ± 10.12 ^c^	14.50 ± 4.43 ^ab^	5.75 ± 4.03 ^a^	21.50 ± 5.51 ^b^	64.00 ± 8.00 ^d^
D90 (µm)	1135.95 ± 46.21 ^a^	1366.08 ± 56.85 ^b^	1367.08 ± 188.91 ^b^	1233.81 ± 197.11 ^ab^	1256.00 ± 97.90 ^ab^	1256.00 ± 97.90 ^ab^
Span	0.79 ± 0.04 ^a^	0.84 ± 0.04 ^ab^	0.90 ± 0.09 ^ab^	0.93 ± 0.14 ^b^	0.82 ± 0.06 ^ab^	0.82 ± 0.06 ^ab^
Protein (%)	7.84 ± 0.32 ^a^	11.48 ± 0.84 ^cd^	10.82 ± 0.73 ^c^	11.67 ± 0.21 ^d^	10.02 ± 0.35 ^b^	9.58 ± 0.27 ^b^
Ash (%)	1.12 ± 0.03 ^bc^	0.97 ± 0.04 ^a^	1.31 ± 0.06 ^d^	1.15 ± 0.02 ^c^	1.14 ± 0.03 ^c^	1.07 ± 0.04 ^b^
Oil (%)	2.26 ± 0.31 ^b^	1.66 ± 0.23 ^a^	2.87 ± 0.51 ^c^	2.59 ± 0.19 ^bc^	2.61 ± 0.26 ^bc^	2.69 ± 0.18 ^bc^
Total starch (%)	81.08 ± 0.28 ^b^	77.82 ± 1.47 ^b^	71.45 ± 3.60 ^a^	79.74 ± 0.21 ^b^	80.85 ± 2.52 ^b^	76.73 ± 0.84 ^ab^
Peak viscosity (cP)	5832.67 ± 281.48 ^c^	3921.50 ± 130.46 ^a^	3834.50 ± 239.24 ^a^	3817.17 ± 398.32 ^a^	3934.50 ± 322.71 ^a^	4498.50 ± 160.54 ^b^
Pt (°C)	73.38 ± 0.52 ^a^	83.38 ± 0.84 ^c^	88.89 ± 0.39 ^d^	87.78 ± 1.59 ^d^	87.26 ± 0.28 ^d^	79.15 ± 6.56 ^b^
Cn (gf)	4301.99 ± 441.02 ^c^	3502.95 ± 262.77 ^b^	3033.92 ± 346.81 ^a^	3044.41 ± 207.56 ^a^	3426.56 ± 221.47 ^b^	3452.29 ± 166.44 ^b^
L*	64.04 ± 0.66 ^a^	64.74 ± 0.39 ^a^	70.04 ± 0.73 ^c^	70.37 ± 0.94 ^c^	66.2 ± 0.36 ^b^	70.74 ± 0.54 ^c^
a*	4.59 ± 0.55 ^d^	5.57 ± 0.32 ^e^	2.05 ± 0.6 ^b^	2.73 ± 0.41 ^c^	7.55 ± 0.39 ^f^	−1.5 ± 0.12 ^a^
b*	27.1 ± 1.79 ^c^	31.94 ± 1.73 ^e^	22.69 ± 2.85 ^b^	22.29 ± 1.58 ^b^	29.69 ± 1.2 ^d^	6.78 ± 0.51 ^a^

Average values with different letters indicate statistical differences (LSD Fisher, *p* < 0.05). P100, hundred-grain weight; TW, test weight; FI, float index; D90, particle size represented by 90% or more of the total particles; Pt, pasting temperature; Cn, consistency.

**Table 2:** Starch hydrolysis traits.

Genotype	RDS (%)	SDS (%)	RS (%)	AUC Index
AX882	70.45 ± 0.49 ^c^	13.97 ± 2.13 ^c^	15.58 ± 1.64 ^b^	108.97 ± 6.29 ^d^
B4	65.74 ± 1.25 ^b^	4.45 ± 1.35 ^ab^	29.81 ± 2.60 ^c^	86.36 ± 2.23 ^a^
BCOT	79.49 ± 1.43 ^d^	5.68 ± 4.44 ^ab^	14.83 ± 3.01 ^b^	101.27 ± 3.99 ^bc^
C4B	55.69 ± 0.34 ^a^	29.73 ± 0.94 ^d^	14.58 ± 0.60 ^b^	107.18 ± 3.43 ^bcd^
C6006	70.56 ± 2.22 ^c^	1.92 ± 0.08 ^a^	27.52 ± 2.31 ^c^	108.21 ± 4.63 ^cd^
C8008	72.09 ± 4.04 ^c^	18.31 ± 4.40 ^c^	9.60 ± 0.36 ^a^	104.45 ± 3.10 ^bcd^
Bread	78.88 ± 0.63 ^d^	8.58 ± 0.74 ^b^	12.54 ± 0.11 ^ab^	100.00 ± 0.20 ^b^

Average values with different letters indicate statistical differences (LSD Fisher, *p* < 0.05). RDS, rapidly digesting starch; SDS, slow-digesting starch; RS, resistant starch; AUC index, index for area under the starch hydrolysis curve considering the value of white bread as 100%.

## Data Availability

The original contributions presented in the study are included in the article, further inquiries can be directed to the corresponding author.
